# Everyday Auditory Environment Among Elderly Cochlear Implant Users

**DOI:** 10.3390/audiolres15060144

**Published:** 2025-10-22

**Authors:** Ulrika Larsson, Ulrika Löfkvist, Karin Hallin

**Affiliations:** 1 Department of Surgical Sciences, Otorhinolaryngology and Head and Neck Surgery, Uppsala University, 751 05 Uppsala, Sweden; ulrika.b.larsson@akademiska.se; 2 Department of Public Health and Caring Sciences, Uppsala University, 751 05 Uppsala, Sweden; ulrika.lofkvist@uu.se

**Keywords:** cochlear implants, aged, social interaction

## Abstract

**Background/Objectives**: For most adults receiving a cochlear implant (CI), the primary goal is to enhance their oral communication with others. The aim of this study was to investigate the total CI usage time per day among retired CI users and to characterize in which auditory environments they were using their CI. One additional aim was to analyze whether usage time, auditory environment, or social factors influenced CI speech perception. **Methods**: Participants completed a questionnaire addressing retirement status, whether they lived with another adult, educational level, and participation in social activities. Speech perception scores were obtained from medical records, and CI datalogging was extracted from the CI programming software. **Results**: Seventy-three CI users aged >65 years were included. The average usage was 12.9 h/day. No statistically significant correlations were found between total usage time or time spent listening to speech and CI speech perception. CI users who regularly met with family or friends had statistically significantly higher CI speech perception than those who did not (*p* = 0.003). **Conclusions**: Social interaction may play a crucial role in supporting speech perception among elderly CI users. Opportunities for communication and participation in social life appear to be important for maximizing benefit.

## 1. Introduction

All individuals receiving a cochlear implant (CI) are to some extent affected by their hearing loss (HL). Some avoid social interactions and no longer use the telephone for oral communication [1]. Oral communication with others, even with a partner and/or children at home, is often disrupted. Many elderly people with HL gradually develop “hearing habits” where they avoid spoken communication because of their hearing difficulties. These habits can be difficult to break and may persist even after hearing is partially restored with a CI, unless specific training is provided [2,3]. For individuals receiving a CI, auditory and communication training constitutes a crucial part of the rehabilitation process. Such training involves systematic practice in everyday listening and oral communication through the CI. In pediatric populations, this is considered essential, and children are routinely offered comprehensive habilitation programs. In adults, however, structured training is less consistently emphasized in clinical practice, likely because most adult CI recipients have already developed spoken language and communication skills [4]. Currently, there are no evidence-based guidelines specifying the intensity or duration of training required to optimize CI outcomes for adults, and the amount of training needed appears to vary substantially across individuals [5]. In fact, a considerable proportion of the variability in speech perception outcomes following cochlear implantation remains unexplained [6]. One potential explanation for this variability could be differences in how much spoken language CI users are exposed to and their own engagement in oral communication with others. As with other complex skills, consistent practice is likely necessary to achieve improvement in the targeted domain. For most adults receiving a CI, the primary goal of rehabilitation is to enhance their ability to engage in listening activities and oral communication with others [7].

In modern CI sound processors, datalogging functions provide information on total daily usage time, as well as time spent in different auditory environments, such as speech, speech in noise, silence, music and noise, and accessory use. Among elderly, retired CI users, a frequent clinical observation and concern is that many spend a large amount of the day in silence. Klein [8] studied the auditory environment in elderly hearing aid users aged 64 to 82 years and found that 40% of total usage time was spent in silence, whereas only 12% was spent speaking or listening to speech. Consistent with these findings, Busch and colleagues [6] studied the datalogging for CI users of different ages and found that adults aged >65 years spent an average of 5.95 h/day in quiet, corresponding to 54% of a mean daily use of 11.3 h. For the same age group, the time spent listening to speech (with or without background noise) was on average 3.34 h/day (30%). Earlier studies have shown that the overall usage time correlates with speech perception outcomes in CI users [6,9,10,11]. In line with these findings, Chow et al. [5] found that adult CI users aged >50 years spent a greater proportion of time in quiet. They also found that older CI users demonstrated greater improvements in speech perception with longer daily device use.

In a review article, Illg and Lenarz [4] described the challenges and opportunities in individualized hearing rehabilitation in the elderly with hearing impairment, which strongly influences reintegration into social life. Almost all studies in their review reported cognitive improvements after CI fitting. Increased social contact and interaction may be possible through CI and may be a possible reason for the improvement in cognitive abilities. CI use could also contribute to working against social isolation and loneliness caused by hearing loss, which can, otherwise, lead to depressive moods. They concluded that the role of the social environment, such as family and friends, should not be underestimated and that further studies are needed to clarify more intensively how comprehensive care for elderly patients can be provided.

The aim of this study was to investigate the total CI usage time per day among retired CI users aged >65 years and to characterize in which auditory environments they are using their CI. In addition, we also investigated if time spent listening to speech was associated with speech perception outcomes with CI and if social factors (living with another adult, participation in clubs or associations, and regular (≥once per week) interaction with family or friends) influenced CI speech perception performance. The Swedish Ethical Review Authority (Dnr 2024-03437-01) approved this study. Written informed consent was given by all included individuals.

## 2. Materials and Methods

**Figure 1:**
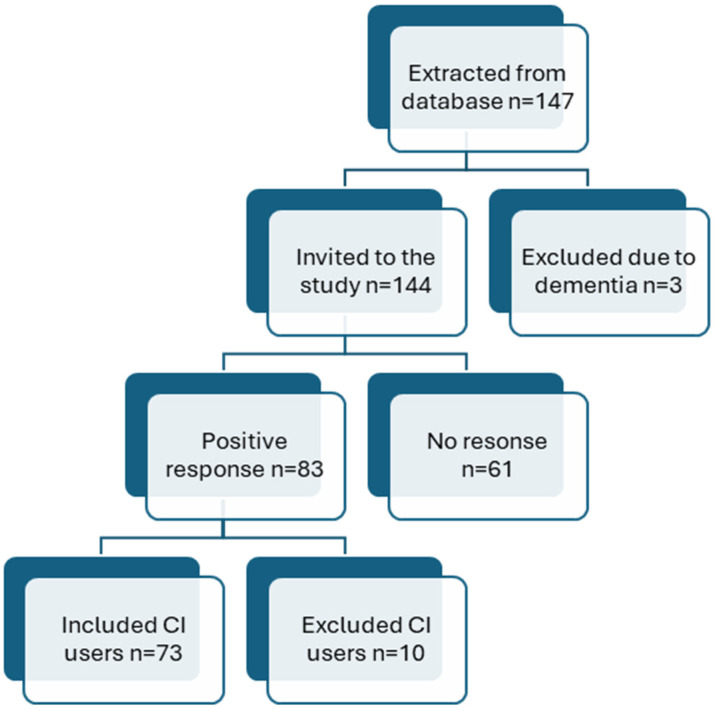
Inclusion process.

CI users aged >65 years, who were retired and using CI sound processor models Nucleus 7 (model number: Cp1000) and 8 (model number: Cp1110) from Cochlear (Lane Cove, NSW, Australia) were identified from our clinical database. These processor models were selected to enable extraction and comparison of auditory environment log data from the sound processor. Each participant used only one CI sound processor. A total of 147 CI users were identified; 3 were not invited to this study due to dementia. Of the 144 CI users invited by mail, 83 responded. Ten respondents were excluded (due to dementia, not being Swedish speaking, or implantation for <1 year), leaving seventy-three CI users for inclusion in this study (Figure 1).

Four of the included CI users were bilateral CI users. One reported regular use of only one implant, while the remaining three used both implants equally. Participants completed a questionnaire addressing retirement status, if they lived with another adult, educational level (no schooling, public school, upper secondary school, or university), participation in social activities (e.g., clubs or associations), and frequency of social interaction (defined as spending time with family or friends at least once per week). Speech perception scores were obtained from medical records, based on the most recent assessment of monosyllabic (MS) word recognition in free field at 65 dB SPL with the CI. On the same date, the datalogging records were extracted from the CI programming software (Custom Sound, version 7.0.090.138; Cochlear, Lane Cove, NSW, Australia). For bilateral users, speech perception performance was calculated as the mean of right and left CI scores.

The sound processor’s datalogging function records average daily usage time. When the CI is active, the sound processor continuously analyzes the acoustic environment and stores the duration spent in each category. The acoustic environments were classified as speech in quiet, speech in noise, quiet, music, noise, or accessory use. Accessory use may include a telecoil, TV streamer, mini-microphone, phone clip, or streaming from a mobile phone. Log data represented the cumulative recording period between two consecutive clinic visits.

IBM SPSS Statistics (version 28) was used for statistical analyses, with a significance level set at an α of 0.05. Linear regression was used to examine whether the acoustic environment, age, social situation (living with another adult, participation in clubs or other associations, and regular (>once a week) interaction with family or friends), or educational level influenced MS word recognition outcomes. The Independent-Samples Mann–Whitney U Test was used to assess if social situation affected time spent across different acoustic environments. The Independent-Samples Kruskal–Wallis Test was used to evaluate if the degree of social interaction (classified as none, one, two, or three positive responses to the social situation variables) was associated with device usage or MS word recognition outcomes. Significance values were adjusted using the Bonferroni correction for multiple comparisons.

## 3. Results

The mean age of the 73 included CI users was 78 years (range: 65–95 years). The CI users included in this study had been implanted for an average of 10 years (range: 1–35 years) at the time of data collection. The standard CI follow-up program for adults at our clinic includes seven appointments during the first year after implantation, followed by one appointment annually at years 2, 3, 5, 7, 9, and so on. During the follow-up program, CI processor fitting and rehabilitation are conducted by the audiologist, the CI engineer, the medical social worker, and the hearing therapist. CI users aged 80 years and older are scheduled for annual visits. Prior to implantation, most CI users had used hearing aids, with an average hearing aid use of 23 years (range: 1–65 years). The responses from the questionnaire, together with CI MS word recognition outcomes, are summarized in Table 1. Datalogging and speech perception data were collected between November 2022 and June 2025. The datalogging period varied between 6 months and 3 years. Data from the sound processor logs are presented in Table 2. The average daily CI usage was 12.9 h (range: 1.7–20.2 h). In Table 2, “use of accessories” includes telecoil, streaming from a mobile phone, other portable microphones, or TV devices. A more detailed description of accessory use is provided in Table 3. Time spent in quiet varied between 0.8 and 17.4 h/day, with an average of 7.2 h/day. On average, 57% of total device usage occurred in quiet (range: 16–96%). Time spent listening to speech or speech in noise (categorized as “live speech”), without the use of accessories, averaged 2.8 h/day (21%; range: 0–69%). When accessory use was included (categorized as “all speech”), assuming accessories were used for speech comprehension, average usage increased to 4.6 h/day (35%; range: 0–81%).

Speech perception with the CI averaged 37% (range: 0–94%). No statistically significant correlations were found between time spent listening to speech (live speech or all speech) and MS word recognition scores (*p* > 0.05), nor between time spent in different auditory environments and speech perception scores (*p* > 0.05).

**Figure 2:**
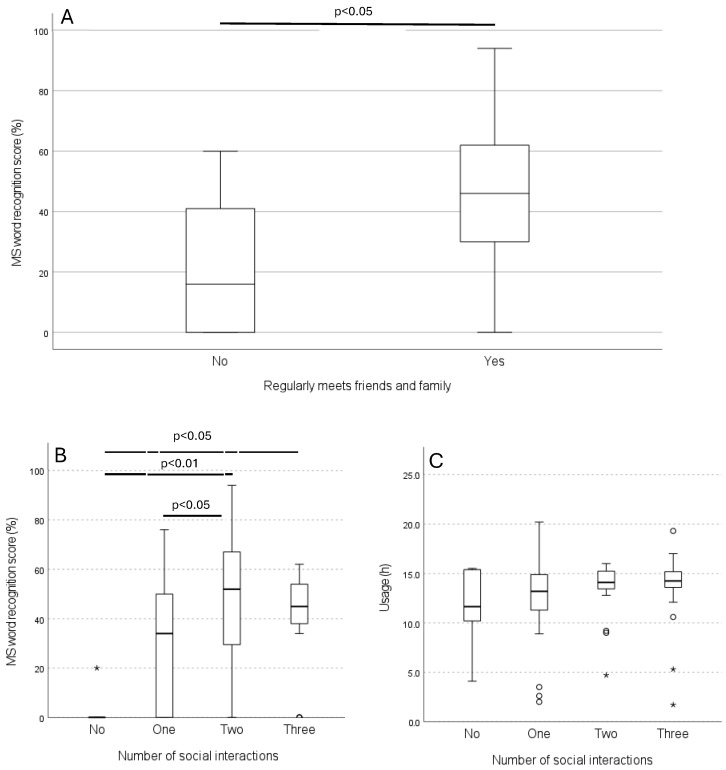
(**A**): Boxplot of MS word recognition scores for CI users who did or did not regularly spend time with friends or family. A statistically significant difference was found between the groups (*p* = 0.003). CI users who spent time with family or friends at least once a week had higher MS word recognition scores (mean: 44%) compared with those who did not (mean: 20%). (**B**): Boxplot of MS speech perception scores by number of social interactions, classified as no, one, two, or three positive responses to questions if they lived with another adult, were involved in social activities in clubs or associations, and regularly (at least once a week) spent time with family or friends. Statistically significant differences were found between no and two interactions (*p* = 0.001), no and three interactions (*p* = 0.021), and one and two interactions (*p* = 0.039). Significance values were adjusted using the Bonferroni correction for multiple comparisons. CI users with more social interactions scored higher on MS speech perception, but no difference was observed between two and three interactions. (**C**): Boxplot of total device usage time by number of social interactions. No statistically significant differences were observed (*p* > 0.05). ◦ and * in the figures indicate outliers and extreme outliers.

Comparing MS word recognition scores among different social contexts showed the following: CI users living with another adult had scores of 38% versus 36% for those living alone; CI users involved in social activities in clubs or associations had scores of 46% versus 33% for those not involved; and CI users who regularly spent time with family or friends had scores of 44% versus 20% for those who did not. CI users who regularly met with family or friends had statistically significantly higher MS word recognition scores than those who did not (*p* = 0.003) (Figure 2A). These CI users also spent statistically significantly more time listening to speech (*p* = 0.035) and noise (*p* = 0.025) than those who did not regularly socialize. No correlations were found between total device usage or age and MS word recognition (*p* > 0.05). Educational level was also not correlated with either MS word recognition scores or total usage time (*p* > 0.05).

The number of social interactions was analyzed, revealing statistically significant differences in MS word recognition scores between CI users with no interactions and those with two interactions (*p* = 0.001) and three interactions (*p* = 0.021), as well as between one and two interactions (*p* = 0.039) (Figure 2B). The average MS word recognition scores were 3% (*n* = 6; range: 0–20%) for CI users with no social interactions, 31% (*n* = 24; range: 0–76%) for one interaction, 50% (*n* = 26; range: 0–94%) for two interactions, and 40% (*n* = 17; range: 0–62%) for three interactions. The number of social interactions did not correlate with total device usage time (*p* > 0.05) (Figure 2C).

## 4. Discussion

Average usage varied hugely among CI users in this study, with a mean of 12.9 h/day. This is consistent with previous studies in this age group [6,11,12]. Alhabib et al. [9], although focusing on children, showed a significant positive correlation between daily CI use and speech perception performance. Lindquist et al. [10] suggested that daily CI use of 12 h/day or more is recommended for optimal outcomes, finding that usage time one month after the first CI fitting predicted speech perception outcomes in adults. Similarly, Holder et al. [11] investigated 300 adult CI users and found a statistically significant correlation between daily usage time and word and sentence recognition, concluding that the highest speech recognition outcomes were associated with daily usage exceeding 10 h/day.

DeFreese et al. [12] investigated the effects of duration of deafness, age at implantation, daily usage time, and speech perception for 614 adult CI users, and found that only daily processor use significantly contributed to the speech perception outcomes. In contrast, our data did not show a correlation between total usage time and speech perception. One possible explanation is that CI users with low usage time may be less likely to participate in studies and, therefore, could be underrepresented in our sample.

Earlier studies on auditory environments for adult hearing aid users showed results broadly similar to ours, although direct comparisons are challenging due to differences in environmental categorizations and study designs. Busch et al. [6] concluded that the types of auditory environments encountered by CI users may depend on age—children typically spend more time in noisy environments and less time in quiet—as well as on country of residence. Klein [8] found that only 12% of time was spent speaking or listening to speech, while Busch [6] found that time spent listening to speech (with or without background noise) averaged 3.34 h/day. In our study, CI users spent 2.8 h/day listening to speech or speech in noise (21%). Time spent in quiet averaged 7.2 h/day (57%), slightly higher than earlier studies of hearing aid users, which reported 27 to 40% time in quiet [8,13]. However, this result is comparable to Busch et al.’s results [6], which reported that senior CI users spent 53% of their total usage time (5.95 h/day of 11.3 h/day) in quiet.

In a study by Eggermont and Vandebosch [14], the elderly (aged 60 and above) were found to watch TV for approximately 4 h/day, with many using TV as a substitute for social interactions or to avoid loneliness. In our study, we cannot determine total TV watching time, but the average usage time of TV-related accessories (a telecoil and TV streamer) was 1.4 h. The time spent watching TV without accessories cannot be determined from our data. One notable observation is that accessories, which can greatly assist CI users when watching TV, attending social gatherings, or using the phone, appear to be underutilized. This suggests that more targeted educational interventions may be needed for this age group to increase confidence and encourage the use of listening devices that could enhance hearing.

While hearing is not the sole component of communication, it plays a central role, and a lack of communication opportunities often leads to social isolation [15]. The Public Health Agency of Sweden has summarized Swedish and international research on the prevalence and consequences of social isolation and loneliness, finding that one-quarter of individuals aged 60 and above experience loneliness. Risk factors for this age group include living alone, loss of a partner, absence of a social network, low social interaction, other health issues, or depression. Social isolation and loneliness were also found to increase the risk of death, independent of the cause [16].

Additionally, studies have shown that regaining access to oral communication and social participation enhances quality of life (QoL) in adults [17,18]. Interestingly, in our data, the only factor that independently influenced MS word recognition outcomes was whether CI users regularly (at least once a week) spent time with friends and family. As expected, these individuals listened to statistically significantly more speech (*p* = 0.035) and noise (*p* = 0.025) compared with those who did not. We also observed that the number of social interactions positively affected MS word recognition scores, with CI users reporting at least two positive social interaction responses, performing significantly better on the speech perception test. These findings support the view that a lack of communication often contributes to social isolation. However, our data cannot determine the direction of this relationship; that is, whether social isolation leads to reduced communication or reduced communication leads to social isolation.

Philpott et al. [7] examined QoL for both CI users and their communication partners (partners, close friends, or caregivers) before and after implantation. They found increased QoL for both CI users and their communication partners after CI. They conclude that the benefit from a CI is not only for the implanted patient but also for their communication partners, whose QoL can improve as a result of reduced caregiver burden, enhanced communication, and greater autonomy of the CI user. They did not find a relation between QoL and CI speech perception. Mäki-Torkko et al. [18] found in their study of QoL among CI users and their “significant others” (partners, close friends, or caregivers) before and after implantation that they experienced a transformation from alienation to a state of normality (not being different from “everyone else”) after CI. Both the CI users and their significant others described a fear, prior to the implantation, regarding the patients’ ability to communicate with others. The fear reduced after the implantation. After implantation, the CI patients managed social situations on their own and took more initiative in communication. After CI, social interactions were more face-to-face and less distant. This led to increased participation and involvement.

### 4.1. Limitations of the Current Study

The sound processor data log provides only the average time spent in different auditory environments and does not provide detailed, time-stamped data. Additionally, precise information regarding how each auditory environment is categorized is not provided [8]. Since daily exposure to sound and verbal interaction through daily activities is expected to be more limited in this age group compared with working adults and children, it would have been beneficial to collect and compare data from age-matched individuals with typical hearing. In this study, we used data logs from a single CI manufacturer (two models of sound processors) to allow comparison between CI users. However, this approach limits the generalizability of our findings, as comparing datalogging across different CI manufacturers is challenging due to differences in categorization and reporting. Furthermore, the speech perception outcome, measured by MS word recognition scores, might not be sensitive enough to find relations relevant to the research questions. A broader measure of outcome, with QoL measures included, might give a more precise result. Other limitations include potential sample bias, as CI users with low device usage may be less likely to participate in studies, which could influence the results. Furthermore, social interaction data were self-reported, which may be subject to recall bias or inaccuracies. Also, health-related issues other than hearing could have influenced their social interactions. Finally, the cross-sectional design of this study prevents determination of causal relationships between social interactions, auditory environment, and speech perception outcomes.

### 4.2. Future Perspectives

Klein and colleagues [8] used the Language Environment Analysis (LENA) system to examine whether hearing aids change the auditory environment of their users. Even though the LENA system was originally developed for children, some studies have validated its use in adults [8,13]. Contrary to their expectations, Klein and colleagues did not find that participants spent more time in speech environments or less time in silence when wearing their hearing aids. This finding supports our prediction that changing long-established habits requires training and support. We are planning a study utilizing the LENA system to assess elderly CI users, with recordings conducted prior to implantation and at three and six months following CI activation. This will provide more detailed insights into how CI use may influence auditory environments over time.

## 5. Conclusions

We found that elderly CI users (>65 years) wore their devices on average 12.9 h/day, with most of that time spent in silence (mean: 7.2 h/day). No correlations were observed between total usage time or time spent listening to speech and MS word recognition score. However, CI users who regularly spent time with friends and family (at least once a week) achieved statistically significantly higher MS word recognition scores. Similarly, the number of social interactions had a positive effect: CI users reporting at least two positive responses to “living with another adult”, “involvement in clubs or associations”, or “regular time with friends and family” demonstrated statistically significantly better MS word recognition outcomes. These findings suggest that social interaction may play a crucial role in supporting speech perception among elderly CI users. Beyond device usage alone, opportunities for communication and participation in social life appear to be important for maximizing benefit. This highlights the potential value of incorporating social support and counselling into rehabilitation programs, as well as encouraging CI users to engage actively in social activities after implantation.

## Figures and Tables

**Table 1:** CI user demographics collected from questionnaire: age, social interactions, educational level, and MS word recognition with the CI.

Age (Years)	Lives with Another Adult (Yes/No)	Educational Level (No School, Public School, Upper Secondary School, or University)	Involved in Social Activities in Clubs or Associations (Yes/No)	Regularly Spends Time with Family or Friends (Yes/No)	MS Word Recognition (%)
Average: 78 Range: 65–95	Yes: 48 No: 25	No school: 1 Public school: 30 Upper secondary school: 20 University: 22	Yes: 26 No: 47	Yes: 53 No: 20	Average: 37 Range: 0–94

**Table 2:** Sound processor log data. Percentages in brackets indicate the percentage of time each CI user spent in each auditory environment.

	Total Usage Time (h)	Speech (h)	Speech in Noise (h)	Quiet (h)	Music (h)	Noise (h)	Use of Accessories (h)
**Average:**	12.9	1.4 (10%)	1.4 (11%)	7.2 (57%)	0.1 (1%)	1.0 (8%)	1.8 (14%)
**Max.:**	20.2	3.9 (26%)	6.6 (47%)	17.4 (96%)	1.1 (7%)	2.8 (18%)	12.7 (73%)
**Min.:**	1.7	0 (0%)	0 (0%)	0.8 (16%)	0 (0%)	0.1 (2%)	0 (0%)

**Table 3:** Sound processor log data for use of accessories.

	Total Use of Accessories (h)	Telecoil (h)	TV Streamer (h)	Mini-Microphone (h)	Phone Clip (h)	Streaming from Mobile Phone (h)
**Average:**	1.8	1.0	0.4	0.0	0.0	0.4
**Max:**	12.7	12.7	6.6	0.8	1.7	4.5
**Min:**	0.0	0.0	0.0	0.0	0.0	0.0

## Data Availability

The data presented in this study are available on request from the corresponding author. The data are not publicly available due to privacy or ethical restrictions.

## Abbreviations

The following abbreviations were used in this manuscript:

CI	Cochlear implant
HL	Hearing loss
MS	Monosyllabic
QoL	Quality of life
